# The Story Behind “A Requirement for Two Cell Types for Antibody Formation *in vitro*”

**DOI:** 10.3389/fimmu.2014.00495

**Published:** 2014-10-17

**Authors:** Donald E. Mosier

**Affiliations:** ^1^Department of Immunology and Microbial Science, The Scripps Research Institute, La Jolla, CA, USA

**Keywords:** cell interactions, antibody response, early career, mentoring, history

This is a personal story about how to get your scientific career started with a bang. It may not be as easy today as it was in the 1960s, but I think the principles still apply. I hope that this personal recounting will be useful to young scientists trying to establish their career.

## Get into a Good Laboratory as Soon as You Can

The way to get your scientific career started is to work with great scientists who are working on important problems in an exciting environment. I spent the summer of 1964 working as a technician in the laboratory of Brigitta Askonas at the National Institute for Medical Research (NIMR) in Mill Hill, a northwest suburb of London. Askonas was a rigorous scientist who was interested in the role of macrophages in the antibody response (1). The job had been arranged by Charles Medawar, a friend during my undergraduate days at Indiana University (IU), and son of Sir Peter Medawar, who was then the Director of the NIMR and a Nobel Laureate for the discovery of immune tolerance. It helped that I had worked in the laboratory of Felix Haurowitz at IU studying rabbit antibody responses to closely related haptens, and thus had some introduction to the field of immunology, and that Professor Haurowitz was friends with Sir Peter. So before I arrived at the University of Chicago to begin medical school in the fall of 1965, I had spent time with three prominent immunologists and I had some sense of the critical issues in the field and felt comfortable at the lab bench. This was just after thymus-derived (T) cells and bone marrow-derived (B) cells had been identified (2), and the cellular requirements for antibody formation were still unknown.

## Find Good Mentors and Do Not Get Discouraged if at First You Do Not Succeed

My first year in medical school was a disappointment. I liked the excitement of a research lab, not the rote learning of anatomy, physiology, and biochemistry. During the spring of 1966, I remember attending a lecture by Don Rowley and being impressed by his enthusiasm and energy. I decided to take a leave of absence from medical school to pursue a Ph.D. (this was prior to the combined M.D./Ph.D. training program). Since Don Rowley and Frank Fitch were in the Department of Pathology, that was my first choice of graduate department. Bob Wissler was then chair of the department and I was assigned by him to a new junior faculty member that left me with lots of lab space and little mentoring. I attempted, at first on my own, to follow up on two seminal technical publications that had appeared in 1963 and 1966. The first was the report by Neils Jerne and Al Nordin (3) that described a method for detecting single antibody-producing cells by a hemolytic plaque assay using sheep red blood cells (SRBC) as the antigen. The second was the report by Bob Mishell and Dick Dutton (4) describing *in vitro* antibody formation against SRBC in mouse spleen cell cultures. My lab notebook from 1966 records many attempts to perfect the Jerne plaque assay, at least some of which were successful, and more attempts to perfect the Mishell–Dutton technique, none of which were successful. I was spending more time talking with Rowley and Fitch trying to solve these problems. They were an ideal team of mentors, with Rowley constantly getting excited about the hypothesis of the day, and Frank Fitch gently guiding the experimental details in his gentlemanly fashion. In January of 1967, they proposed the brilliant idea of sending me to Dutton’s lab at the Research Institute of Scripps Clinic (now renamed as The Scripps Research Institute, where I have been since 1992) in La Jolla, CA, USA. A trip from midwinter Chicago to sunny La Jolla seemed like a great idea to me. Bob Mishell and Dick Dutton were very cordial hosts (Dick Dutton is a lifelong friend), and I learned all the secrets of the culture technique that were not revealed in their paper, such as, only selected sources of SRBC and fetal calf serum would work, the rabbit complement for developing the hemolytic plaques needed to come from the right rabbit, and adding new medium to the cultures each day was essential. They also showed me their plastic culture boxes that were much easier to use for the required gas mixture than the desiccator jars that I had been using in Chicago (I still have several of these culture boxes in my lab these many years later). I was able to take this information back to Chicago and finally achieve success by mid-March of 1967, with literally hundreds of plaque-forming cells (PFC) per million mouse spleen cells cultured.

## Ask Important Questions

One of the critical questions in immunology was the role of “macrophages” in the antibody response. Several papers had suggested that incubation of macrophages with antigen enhanced the immune response when the mixture was injected into animals, but their role in promoting antibody formation was unknown at the time. There were some crazy (in retrospect) theories floating around, like RNA from macrophages instructing B cells to make antibody (better not cited). This was long before we knew how antibody variability was generated, and instructional theories of antibody folding favored by Felix Haurowitz still had many proponents. The new Mishell–Dutton culture technique provided the opportunity to address the role of macrophages *in vitro* since inspection of the tissue culture dishes by phase microscopy (I was lucky to be in a pathology department) clearly showed large, adherent phagocytic cells that were often surrounded by loosely or non-adherent mouse splenic lymphocytes^1^. It was known at the time that macrophages would adhere to glass (5), but adherence to plastic culture dishes was a novel observation. My lab notebook shows several experiments where spleen cells were separated into “sticky” and “non-sticky” cells, then later revised to “adherent” and “non-adherent” cells, and a final nomenclature evolution to “macrophage-rich” and “lymphocyte-rich” during the preparation of my 1967 manuscript (6). It was apparent by late April of 1967 that neither “macrophage-rich” or “lymphocyte-rich” spleen cell subpopulations alone could respond to SRBC, but adding SRBC to the “macrophage-rich” subpopulation for 30 min prior to recombining with the “lymphocyte-rich” subpopulation allowed a robust PFC response that was comparable to intact spleen cells. Experiments were numbered sequentially in my notebook, and the productive replicate experiments in April to June 1967 were numbered 29–36, each involving 4–5 days of culture before the Jerne PFC assays. For young scientists starting out, it is worth noting that most of the first 25 experiments performed over a period of 8 months were not productive, so be patient, seek help, and collaborate with experts.

## Accept Help Graciously

Speaking of large numbers of experiments, there were also large numbers of manuscript drafts over the summer of 1967 before the final version was submitted to Science in October. This was my first paper, and I spent many hours with Don Rowley refining each word in the manuscript, shortening it to reach the word limit for Science, hand drawing Figures 1 and 2 in India ink (I still have the faded original Figure 1 in my notebook), and refining the technical notes that would now appear as supplemental information but were then included in the references. Frank Fitch also reviewed the manuscript, but it was Don Rowley who insisted that it meets his high standards – the 30th revision finally made the cut. After all this effort, I was amazed that both Don Rowley and Frank Fitch insisted that they should not be co-authors. This was generous by the standards of the day, and would be next to impossible in the current climate of underfunded scientists scrambling for NIH grants.

Subsequent work that I performed in Don Rowley’s laboratory suggested that the cell required for the SRBC antibody response in the “macrophage-rich” subpopulation was quite rare (7) and unlikely to be the predominant macrophage. This led to a reversion of the nomenclature to “adherent” cell, and later work by Ralph Steinman identified the key adherent cell to be the dendritic cell. Once again, Rowley and Fitch declined to be co-authors on my paper, but the mathematician who was familiar with limiting dilution analysis, Lionel Coppleson, was appropriately acknowledged. In 1969, I was convinced to return to medical school, but I continued to work part-time in the Rowley lab with fellow students Jeffrey Roseman, Lee Leserman, and Bob Waterston. Great times in a great laboratory! When I finished medical school, I went back to Mill Hill for a short but intense post-doc training with Avrion Mitchison, where I met Martin Raff and Harvey Cantor, and enjoyed many hours of discussion and a few hours at the bench.

In retrospect, my first paper was one of my best, and helped establish the concept that antibody production by B cells was dependent upon cell cooperation between antigen-presenting cells and the responding lymphocytes. Subsequent work (7, 8) showed that T helper cells were also required for the antibody response, with the last paper finally including Frank Fitch and Don Rowley^2^ as co-authors. This requirement for three cell types had been predicted by the limiting dilution experiments (7), and led to much subsequent work by many immunologists on antigen-processing, helper T cell function, and the genetic basis for antigen recognition by T cells and B cells.

## Conflict of Interest Statement

The author declares that the research was conducted in the absence of any commercial or financial relationships that could be construed as a potential conflict of interest.

## Footnotes

^1^Rowley and Fitch had been studying antibody responses in rats *in vivo*, but we were never able to adopt the Mishell–Dutton technique to work with rat spleen cells in culture.

^2^Sadly, Donald Adams Rowley died in February of 2013, and I was one of many of his former students who gathered in Chicago to pay tribute to him last spring. This article is dedicated to him, a generous and skilled mentor whose contribution to the start of my career was unique and is appreciated more with each passing year.
